# The Controversial Role of Vitamin D in Thyroid Cancer Prevention

**DOI:** 10.3390/nu14132593

**Published:** 2022-06-23

**Authors:** Ana Palanca, Francisco Javier Ampudia-Blasco, José T. Real

**Affiliations:** 1 Endocrinology and Nutrition Department, Valencia University Clinic Hospital, 46010 Valencia, Spain; jose.t.real@uv.es; 2 INCLIVA Biomedical Research Institute, 46010 Valencia, Spain; 3 CIBERDEM, CIBER Diabetes and Associated Metabolic Diseases, 28029 Madrid, Spain; 4 Department of Medicine, Medicine Faculty, University of Valencia (UV), 46010 Valencia, Spain

**Keywords:** vitamin D, thyroid cancer, prevention, anti-tumorigenic

## Abstract

Thyroid cancer is the most common endocrine malignancy and exhibits rising incidence. Annual incidence varies by sex, age, and geographical location. It has been reported that impairment of vitamin D signalling promotes thyroid cancer progression. Recent studies have shown that vitamin D, a fat-soluble vitamin that acts as both a nutrient and a hormone, may have utility in the prevention of autoimmune thyroid-related diseases. However, the precise role of vitamin D in the pathobiology of thyroid cancer is controversial. Previous studies have suggested that elevated serum vitamin D levels have a protective role in thyroid cancer. However, there is also evidence demonstrating no inverse relationship between vitamin D levels and the occurrence of thyroid cancer. Furthermore, recent data provide evidence that circulating vitamin D concentration is inversely correlated with disease aggressiveness and poor prognosis, while evidence of an association with tumour initiation remains weak. Nevertheless, a variety of data support an anti-tumorigenic role of vitamin D and its potential utility as a secondary chemopreventive agent. In this review, we highlighted recent findings regarding the association of vitamin D status with the risk of thyroid cancer, prognosis, potential mechanisms, and possible utility as a chemopreventive agent.

## 1. Introduction

Thyroid cancer incidence has been increasing significantly across the globe for the past few decades, largely because of growing cases of papillary thyroid cancer [1]. The incidence of more advanced forms of thyroid cancer have also augmented [1]. This has not been entirely due to surveillance and diagnosis advancements, as true increases in thyroid cancer morbidity have also been reported [1,2].

Studies over these decades, including a recent meta-analysis [3], have indicated an association of lower levels of 25-hydroxyvitamin D in the serum with risk of thyroid cancer. However, this has been controversial with other studies suggesting a lack of such an association [4]. Vitamin D is a fat-soluble prohormone that plays an undisputed role in maintaining calcium and phosphorus homeostasis, thereby preserving bone health [5]. In addition, vitamin D can also modify immune function, cell proliferation, differentiation, and apoptosis, all of which are related to neoplastic growth and tumorigenesis [6].

Supplementation with vitamin D has been suggested as a cancer preventive strategy. Vitamin D, which is naturally synthesized upon sunlight exposure or acquired through the diet, is converted to 25-hydroxyvitamin D/25(OH)D in the liver and 1,25-dihydroxyvitamin D/1,25(OH)_2_D/calcitriol, the active biological form, in the kidneys [7]. Deficiencies in either source may contribute to inadequate vitamin D levels. Although standard thresholds for vitamin D deficiency are debatable, recent studies have suggested a prevalence of deficiency (<50 nmol/L 25(OH)D) of 40% in the United States (US) [8] and up to 93% among South Asian people living in the United Kingdom [9]. Given these high rates of deficiency in some populations and the potential association of lower vitamin D serum levels with risk of thyroid cancer, it is of scientific and clinical interest to explore this association, potential cancer-related mechanisms of action of Vitamin D, and the evidence of its supplementation as a cancer preventive therapy.

In this review, we summarized the relationships of vitamin D insufficiency and supplementation with risk of thyroid cancer. Further, we discuss potential molecular mechanisms of anti-neoplastic activity of vitamin D.

## 2. Epidemiology of Thyroid Cancer

Thyroid cancers stem from two types of parenchymal thyroid cells, the follicular cells, which line the colloid follicles, synthesising thyroid hormones, and the parafollicular cells or C cells, which produce calcitonin [10]. Follicular cell-derived thyroid cancers account for more than 95% of all cases, which include well-differentiated papillary and follicular carcinoma in its large majority [11]. Thyroid cancer usually develops gradually through carcinogenesis and genetic alterations in genes, such as those involved in mitogen-activated protein kinase (MAPK) or the PI3K/Akt pathways [10]. Such alterations promote the proliferation of follicular cells and lead to a malignant phenotype with the ability to invade neighbouring tissues and metastasise to distant body organs [11]. The large majority of thyroid cancer cases are identified as small papillary thyroid carcinomas [10]. Rising incidence rates over the last few decades have been largely due to increases in cases of the papillary pathologic subtype [1]. It has been shown that incidence rates are two-fold lesser in middle-income countries compared to those with high income [12]. This was found to be true both in men (1.40/100,000 versus 3.60/100,1000, respectively) and women (4.70/100,000 versus 11.10/100,000, respectively). On the other hand, in the US and Australia, rates had been declining prior to 1990 [12]. Increasing rates in those countries since then may be related to diagnostic practices rather than just to a true increase in thyroid cancer incidence. In a global analysis, the mortality rate was 0.3 per 100,000 men and 0.6 per 100,000 women [12].

As the fastest growing cancer in incidence, thyroid cancer was previously on course to be the fourth most common cancer in the US by 2030 [13]. However, incidence has recently stabilized because of the incorporation of more conservative practices in the diagnosis of thyroid cancer [14]. This adjustment was made because of an increase in the diagnosis of non-aggressive and clinically less severe tumours. In addition, mortality rates for thyroid cancer are apparently levelling off or decreasing, especially in most of the previous higher-mortality countries, presumably because of better treatment of the disease and enhanced diagnosis and management [12,14].

Vitamin D deficiency is globally widespread regardless of latitude and human development index [15]. A systematic review based on WHO data on vitamin D status in low and middle-income countries (LMIC) reported that, in some LMIC analysed, vitamin D deficiency was a public health concern, but not in all [16]. However, representative population-level 25(OH)D data are limited or missing in most LMIC, thus hampering efforts to assess vitamin D status in those countries. A previous multi-national study estimating the prevalence of vitamin D deficiency in Europe suggested that vitamin D deficiency was also widespread within different European countries, although there was considerable variation in ethnicity, age, latitude, and prevailing weather [17]. Conclusions regarding whether vitamin D deficiency plays a causal role in this relationship to thyroid cancer incidence cannot be drawn from these population-based studies. Other nutritional and environmental factors, such as exposure to xenobiotics or living in volcanic areas, among others, are also to be considered [18].

## 3. Relationship of Vitamin D Levels with Thyroid Cancer Prognosis and Aggressiveness

Exposure to ionizing radiation, chemical genotoxins, and obesity have been linked to an increased risk of thyroid cancer [10]. Thyroid neoplasia is associated with genomic instability enhanced by risk factors, causing early heritable differences (e.g., RET/PTC and PAX8-PPARγ rearrangements, BRAF and RAS point mutations) and native activation of thyrocyte-signalling pathways [19]. In addition to these risk factors, a low serum level of 25-(OH)D has been suggested by many studies to be a risk factor for thyroid cancer. In this section, we summarize recent evidence for and against this association.

In an earlier pilot study conducted in the US, no associations were found between low vitamin D levels and thyroid cancer diagnosis [20]. Likewise, vitamin D status was not found to be associated with clinical stage or other prognostic variables [20]. Stepien et al. investigated levels of vitamin D metabolism by assessing 25(OH)D and 1,25(OH)_2_D in cases of thyroid cancer compared to a healthy control group [21]. Concentrations of 1,25(OH)_2_D significantly correlated with cancer clinical stage, with stage 4 having the lowest concentrations [21]. Conversely, a recent study found that low vitamin D was not associated with characteristics of aggressiveness such as multicentricity, lymphovascular invasion, and metastases in papillary thyroid cancer [22], highlighting the continued controversy in this field. Laney et al. also estimated differences in the frequency of vitamin D insufficiency between thyroid nodular goitre and cancer cases [23]. They found vitamin D insufficiency to be associated with metastatic growth.

Recent studies uphold the potential that serum 25(OH)D levels contribute to the risk of thyroid cancer. A 2019 meta-analysis of 14 case-control studies determined that serum 25(OH)D was lower in thyroid cancer prior to thyroidectomy [3]. The association did not extend to patients who had undergone a thyroidectomy. The authors concluded that vitamin D deficiency may be a thyroid cancer risk factor. A limitation of this study was that measurement methods and thresholds for serum 25(OH)D differed between eligible studies. A recent multi-institution study of thyroid cancer cases and controls in China found a significant protective effect of both 25(OH)D and vitamin D binding protein against thyroid cancer [24]. Other recent studies, however, contribute to the controversy of an association with thyroid cancer risk. Kuang et al. recently compared papillary thyroid cancer cases to benign thyroid disease cases and found no association of circulating 25(OH)D with malignancy or prognosis of papillary thyroid cancer [25].

Vitamin D activity is understood to contribute to the physiological health of the mammary gland [26], and low levels of circulating 25(OH)D have been previously suggested to be correlated with the risk of breast cancer [27]. However, a recent meta-analysis of supplementation found no protective effect for breast cancer [28]. In most breast cancer cells, the expression of the mitochondrial enzyme CYP24A1, which regulates the catabolism of the active form of vitamin D, is increased [26].

Genetics and gene expression activity in the vitamin D pathway have also been implicated in thyroid cancer. In addition to increased expression of the vitamin D receptor (VDR) (responsible for mediating 1,25(OH)_2_D cellular actions) in papillary thyroid cancers, activity of the gene encoding CYP24A1 has also been found to be increased [29]. The association of high CYP24A1 also extended to lymph node metastasis, vascular invasion, and tumour size. Adding to the controversy over the role of vitamin D in cancer, these findings are converse to the notion that low-serum vitamin D is associated with increased aggressiveness and poor prognosis of thyroid cancer, as discussed in subsequent sections. However, the findings of high VDR expression were related to the tumour versus benign tissues and did not explore the relationship of vitamin D activity or VDR with tumour progression or prognosis. A more recent study also identified elevated VDR protein and mRNA expression in papillary thyroid carcinoma compared to healthy and benign thyroid tumours [30]. Higher CYP24A1 expression in papillary thyroid carcinoma was also found but did not reach significance, although the sample was small. 

Interestingly, polymorphisms in vitamin D pathway-related genes have been shown to be associated with risk of breast cancer, specifically the *FokI* variant of VDR that confers risk when it coincides with other breast cancer risk genotypes [31]. The understanding of the role of vitamin D in colorectal cancer is better established with strong evidence that circulating 25(OH)D is protective [32]. A proposed mechanism of action is the VDR-dependent regulation of the Wnt/β-catenin pathway [33]. Whether similar mechanisms are at play in thyroid cancer remains a subject of investigation. Associations between some tumour types and specific single nucleotide polymorphisms (SNPs) within the VDR gene have been investigated. Named VDR SNPs that have been evaluated include *Fok1*, *Bsm1*, *Taq1*, and *Apa1* [34]. While meta-analysis has not been supportive of associations of these variants with the risk of some cancers, including prostate [35], studies have supported their association with disease aggressiveness. For example, the T allele and TT genotype of *FokI* have been shown to correlate with papillary thyroid cancer aggressiveness, making this genotype a potential prognostic indicator for this cancer [36].

## 4. Potential Anti-Neoplastic Functions of Vitamin D

**Figure 1:**
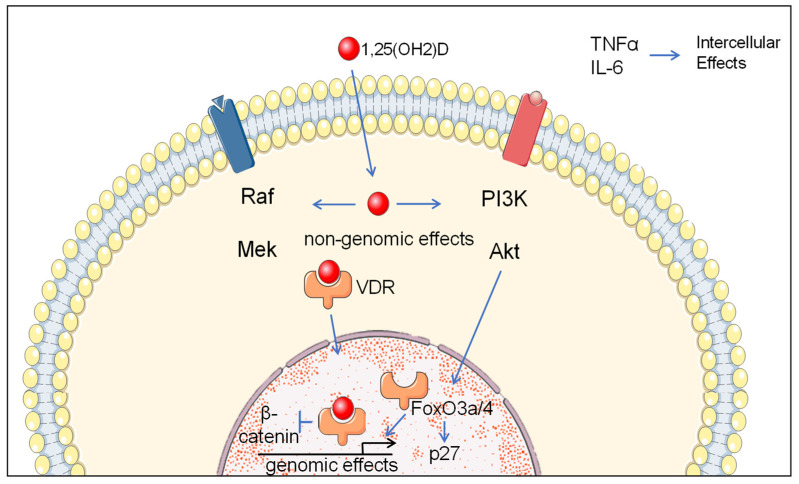
Vitamin D-regulated pathways in cancer. Cancer-related intracellular non-genomic and genomic mechanisms of molecular pathway regulation by vitamin D and intracellular molecular factors regulated by vitamin D. Non-genomic activation of PI3K and MAP kinases occurs downstream of ligand-bound VDR. FoxO3a/4 signalling and the expression of p27 cyclin-dependent kinase accumulation can result from vitamin D/VDR activation of PI3K signalling. Genomic effects involve regulatory effects of nuclear ligand-bound VDR at responsive gene elements. Nuclear ligand-bound VDR also inhibits β-catenin signalling. Decreased TNFα and IL-6 signalling induced by vitamin-D can have intercellular effects by inhibition of pro-tumorigenic inflammation.

Vitamin D is a secosteroid that regulates a large number of genes through activation of the VDR transcriptional factor [37]. Vitamin D directly or indirectly regulates multiple signalling pathways involved in cellular proliferation, apoptosis, inflammation, invasion, angiogenesis, and metastasis (Figure 1) [6]. Non-genomic activation of PI3K and MAP kinases can occur downstream of ligand-bound VDR [38]. It has also been documented that vitamin D is a modulator of microRNA expression and cancer stem cell biology [39]. This includes tumour-suppressive microRNAs miR-22, miR-296-3p, and miR-498 [40]. Vitamin D deficiency has been associated with many health conditions, including rickets in children, osteomalacia in adults, fractures, cancer, immune effects (tuberculosis, respiratory tract infections, asthma, atopic dermatitis), hypertension, heart disease, and dementia [41]. Low plasma concentrations of vitamin D have also been associated with thyroid-related diseases, such as Hashimoto’s thyroiditis, an autoimmunity-mediated disease that can cause hypothyroidism and the most common type of thyroiditis [5]. Deficient vitamin D signalling has been reported to promote thyroid tumorigenesis [42].

Serum 25(OH)D, the primary circulating form of vitamin D, is considered to represent the reserve level of vitamin D in the human body. Aside from regulating calcium and phosphorus metabolism and bone absorption, vitamin D affects cell differentiation and proliferation [6]. Vitamin D and its metabolites, 1,25(OH)_2_D and vitamin D receptor (VDR), are closely related to tumour occurrence, development, and prognosis [43]. Nevertheless, the role of vitamin D in carcinogenesis remains controversial. Potential anti-tumorigenic mechanisms of vitamin D are described in Table 1.

Previous studies have not confirmed a negative correlation of vitamin D levels with thyroid cancer risk [44,45,46]. Conversely, many studies, including the above-mentioned meta-analysis, support a contribution of vitamin D deficiency to thyroid cancer [3]. Indeed, the pathogenesis of many other cancers has been proposed, such as breast, colon, prostate and pancreas [47]. Still, potential anti-neoplastic mechanisms of vitamin D are not well understood or defined. Direct binding of activated vitamin D to VDR and indirect interaction with other transcriptional regulators or cell signalling systems with anti-neoplastic potential have been implicated, supporting the role of vitamin D in thyroid cancer [6,42]. VDR forms heterodimers with retinoid X receptor (RXR). The heterodimer binds and regulates specific genetic elements. Binding of the active form of vitamin D, 1,25(OH)_2_D, to VDR causes the exchange of corepressors with coactivators in the VDR-RXR complex, thereby supporting target gene transcription [48]. Vitamin D responsive elements in target genes drive altered transcription in response to vitamin D, depending on the cell type [37,49].

The cell cycle can be arrested by activated vitamin D in the progression from the G1 phase to the S phase, inhibiting the growth of tumour cells [42]. Moreover, activated vitamin D can induce apoptosis in colorectal adenoma and carcinoma cells, murine squamous cell carcinoma cells, and hepatocellular carcinoma cells by activating pro-apoptotic proteins and inhibiting anti-apoptotic proteins [50,51,52]. Previous studies have found that inflammatory cell infiltration was noted in thyroid cancer, and further research found that these inflammatory cells may be pro-tumorigenic [53]. It has been reported that the inflammatory response can be reduced by activated vitamin D [54]. Pituitary transcription factor-1 (Pit-1), which regulates differentiation, proliferation, and apoptosis, is transcriptionally overexpressed in breast cancer, and its expression is inhibited by 1,25(OH)_2_D, presenting a possible antineoplastic mechanism [55]. In well-differentiated human thyroid carcinoma cells (TCP-1 papillary thyroid carcinoma, WRO follicular carcinoma, and FRO anaplastic carcinoma cells), dose-dependent antiproliferative effects of vitamin D (G1 arrest and nuclear accumulation of p27) have been observed [56,57]. This effect was mediated by both PTEN/Akt-dependent and independent mechanisms. The PTEN/AKT pathway constitutes an important pathway regulating signalling that affects multiple biological processes, such as apoptosis and cell proliferation. The activity of PTEN, a well characterised tumour suppressor, is lost in many primary and metastatic human cancers [58]. The menin transcriptional activator interacts with VDR [59]. In multiple endocrine neoplasia type 1 (MEN1), VDR target gene transcription was reported to be regulated by this pathway and to be significantly lower than in normal tissues [59]. An et al. demonstrated, in a panel of head and neck squamous cell carcinoma cell lines, that cell cycle arrest by 1,25(OH)_2_D was dependent on FoxO3a and FoxO4, which are bound and regulated by VDR [60]. Vitamin D has been implicated in the balance between autophagy and apoptosis. In hepatocellular carcinoma, deficiency in vitamin D was found to be correlated with lower levels of both autophagy marker LC2 and pro-apoptotic caspase-3 [52]. In murine squamous cell carcinoma cells, active vitamin D was found to promote apoptosis through the upregulation of MEK kinase-1 (MEKK-1) and increased caspase-dependent cleavage of mitogen-activated protein kinase (MEK) [51]. Differentiation, accompanied by decreased proliferation and survival, was shown to be promoted by 1,25(OH)_2_D in a variety of carcinoma cell lines, including 8305C and 8505C anaplastic thyroid cancer, W480-ADH human colon carcinoma, and MDA-MB-453 human breast carcinoma cells [61,62]. Antagonism of EGF and Wnt/β-catenin pathways was found to result in epithelial-mesenchymal transition in these tumour cells. Depending on the cellular context, VDR may directly bind β-catenin and compete for the binding of β-catenin or the transcriptional coactivator p300 with transcription factors TCF/LEF (T-cell factor/lymphoid enhancer factor) [63]. Alternatively, co-repressor recruitment to TCF/LEF may mediate repression [63].

The effect of vitamin D insufficiency on carcinogenesis is also controversial. Vitamin D metabolites have been shown to have considerable chemopreventive activity in preclinical models. For example, the combination of vitamin D and indomethacin was robustly chemopreventive in a rat model of colon cancer driven by carcinogenesis [64]. This treatment also abrogated the increased carcinoembryonic antigen (CEA) and platelet-derived growth factor (PDGF) in carcinogenic-induced tumours. Mechanisms for chemoprevention by vitamin D are still unclear.

Immunologic effects of vitamin D are an attractive research target in the study of the anti-neoplastic activity of vitamin D. In fact, VDR is expressed in distinct kinds of inflammatory cells, and vitamin D is capable of inhibiting chronic inflammation and consequent immune cell proliferation, which play important roles in cancer outcomes in a variety of tumour types [65]. Vitamin D was shown to increase antitumorigenic CD8+ T-cell infiltration in breast tumours [66]. Vitamin D may also inhibit pro-tumorigenic inflammation through the down-regulation of mediators, including cytokines TNF-α and IL-6 [67].

Overall, there is evidence that the anti-neoplastic effects of vitamin D include but are not limited to increased apoptosis, arrested cell cycle, inhibition of proliferation, differentiation, reduced inflammatory response, and decreased invasiveness [68]. Clearly, the mechanisms by which vitamin D exerts anti-neoplastic effects are not entirely understood and require further study, including clinical and basic research.

## 5. Vitamin D Supplementation and Thyroid Cancer Prevention

Studies of epidemiologic factors, including geography, latitude, daily sunlight exposure, lifestyle patterns, and behaviour, have indicated a protective role of vitamin D against tumours [69,70,71,72]. Notably, incidence and mortality rates of cancers including melanoma, lymphoma, prostate, colorectal, ovarian, kidney, oesophagus, stomach, and breast cancers have been linked to low Vitamin D levels [73,74,75,76,77].

Ultraviolet light (UVB)-dependent dermal synthesis is the main source of vitamin D [39]. Vitamin D is of limited prevalence in the diet, but it exists as ergocalciferol (vitamin D2) in plants and cholecalciferol (vitamin D3) in animals [39]. A 2008 study showed that excess exposure to sunlight was associated with skin cancer but inversely associated with the risk of other malignancies [78]. It was observed as early as 1941 that people in northern latitudes in the US were more prone to dying of cancer than people living in southern states [79]. Published work following this observation was not seen until the 1980s. Garland reported that mortality from colon cancer was higher in people from the north-eastern US compared to people from the southern US [80,81]. This finding paved the way toward further knowledge on the role of Vitamin D in other types of cancers [82,83].

Based on these observations and demonstrated associations of vitamin D levels with cancer risk, supplementation of vitamin D has been investigated as a preventive measure against cancer. Several randomized, controlled studies have been conducted, although they vary in the dose of vitamin D supplementation. A recent meta-analysis of these studies showed achievement of increased 25(OH)D concentrations in circulation and a significant reduction in cancer mortality with vitamin D supplementation [84]. No difference in incidence of cancer was found.

Again, controversy prevails in the study of vitamin D supplementation for cancer prevention. Another large meta-analysis and systematic review of randomized controlled trials covering over 18,000 participants in 2018 found no protective effect of vitamin D supplementation on cancer incidence [85]. This study covered several tumour types, and its limitations included short follow-up time in the majority of included studies and lack of incidence and mortality as primary outcomes in individual studies. Whether there is efficacy of vitamin D in preventing tumour progression and improving prognosis as a secondary chemopreventive therapy is an interesting possibility that was not addressed by this study.

Despite a large number of trials testing the role of vitamin D supplementation in bone and other health conditions, evidence remains inconclusive. Clinical trials assessing the effects of vitamin D supplementation have yielded conflicting results, presumably due to poor study design in many cases and different study populations [86]. A recent review testing vitamin D supplementation in breastfeeding infants reported that supplementation reduced vitamin D deficiency, although insufficient evidence was available to determine its effects on bone health [87]. A Cochrane systematic review including 53 RCTs and 91,791 older individuals found no reduction in the risk of fractures with vitamin D supplementation alone, whereas, in combination with calcium, the risk of fractures was moderately reduced [88]. Another randomized controlled trial covering over 5000 healthy adults, found that high-dose vitamin D supplementation did not prevent falls or fractures in this vitamin D-sufficient population [89]. Conversely, there is evidence supporting a reduction in falls with vitamin D supplementation among older individuals with poor vitamin D status [90]. Vitamin D supplementation has also been found to be modestly beneficial in the prevention of osteoporotic fractures in older people at risk of vitamin D deficiency [91]. Additionally, available data support the role of vitamin D supplementation in reducing the risk of acute respiratory infections and the frequency of asthma exacerbations [92]. In regard to cardiovascular health, low vitamin D levels have been linked to an increased risk of cardiovascular events. However, robust clinical evidence supporting the role of vitamin D supplementation in the reduction of the risk of cardiovascular events is missing [93]. Data have also indicated an inverse relationship between vitamin D levels and the risk and severity of multiple sclerosis. A meta-analysis in 2018 did not find therapeutic effects of vitamin D in multiple sclerosis patients [94]. However, more recent clinical trials testing high-dose vitamin D showed some protective effects on MRI lesions in patients with low vitamin D levels [95]. In sum, although there seems to be some benefit for a broad spectrum of health conditions, mostly among vitamin D-deficient individuals, results from clinical trials are still heterogeneous and inconsistent [96]. A need arises for further high-quality research defining the role of vitamin D supplementation and identifying those individuals who would benefit the most.

## 6. Discussion and Conclusions

Association studies have been controversial, and their results have not been consistent. Consistency in measurement methods, thresholds, and population characteristics may improve the agreement between studies. Although previous studies have suggested an association of low vitamin D status with thyroid cancer, more recent and robust studies tend to suggest weak or no association with incidence, but significant association with prognosis. Low vitamin D levels in patients with thyroid conditions may be explained by low vitamin D intake, malabsorption, or lack of sun exposure. Vitamin D status may likely represent a general marker of good health. A young, healthy adult of average weight with good lifestyle habits is more likely to have higher 25(OH)D levels and a lower risk of chronic disease [97].

The studies reviewed above suggest that circulating vitamin D concentration has an inverse relationship with various types of cancer, such as breast, prostate, and colorectal cancer. Environmental and genetic factors play a significant causal role in both cancer and vitamin D deficiency. This makes a mechanistic link between vitamin D and cancer risk elusive. However, many sources of evidence, including association studies showing an inverse correlation of vitamin D with cancer mortality, observations of seasonal and geographical differences in cancer mortality that correlate with sunlight exposure and vitamin D levels, and genetic variants in VDR and related genes that confer risk of cancer strongly, indicate a role of vitamin D in cancer biology and prevention.

Several targets in the vitamin D pathway have been identified in thyroid cancer, such as VDR and CYP24A1. An effect of vitamin D on outcome and disease aggressiveness is evident in thyroid cancer, although prognostic testing based on this has not been developed. The case for vitamin D supplementation as a preventive seems justified, if not to prevent the initiation of disease, then to prevent disease progression as a secondary chemopreventive. The appropriate dosage of vitamin D as a chemopreventive agent in thyroid cancer is also unresolved. Observational and controlled clinical studies are needed to assess the hypothesis that vitamin D can prevent thyroid cancer or thyroid cancer progression. Serum-based trials may be utilized to standardize optimal 25(OH)D thresholds and vitamin D dosage required to achieve optimal levels.

A clinical vitamin D equivalent should be designed for optimal anti-cancer effects and to avoid hypercalcaemia due to increased vitamin D uptake and toxicity. Interestingly, vitamin D has been combined with certain anticancer drugs to achieve additive and even synergistic anti-tumour effects [98]. While further validation of the role and mechanism of vitamin D in cancer progression is required, clinical evaluation of this association and the utility of vitamin D as a cancer treatment and/or preventive is well justified.

With vitamin D deficiency so common worldwide, its potential effects on tumour incidence and mortality are important. While associations of vitamin D levels with cancer incidence are controversial, a correlation with tumour aggressiveness and mortality are more evident. This is true for thyroid cancer and several other cancer types. Several sources of evidence, including correlations of sunlight exposure with cancer mortality and genetic linkage of VDR and related genes with cancer risk and mortality, further provide credibility to the hypothesis that vitamin D is protective against cancer. Rigorous clinical evaluation is still necessary to solve this question and to standardize optimal vitamin D levels and appropriate dosing to successfully impact disease outcomes in thyroid and other cancers.

## Figures and Tables

**Table 1:** Potential anti-tumorigenic mechanisms of vitamin D.

Effect	Molecular Determinants	Mechanism	Publications
Anti-proliferation	P27, PTEN, Akt Fox O3a	G1 arrest Cell cycle arrest	[58] [61]
Apoptosis	MEK cleavage MEKK-1	Apoptosis	[52]
Differentiation	EGF, Wnt/β-catenin	Epithelial-mesenchymal transition	[63]
Immunologic	TNF-α and IL-6	CD8+ T cell infiltration Anti-inflammation	[66] [67]

## References

[1] Seib C.D., Sosa J.A. (2019). Evolving understanding of the epidemiology of thyroid cancer. Endocrinol. Metab. Clin. N. Am..

[2] Morris L.G., Tuttle R.M., Davies L. (2016). Changing Trends in the Incidence of Thyroid Cancer in the United States. JAMA Otolaryngol. Head Neck Surg..

[3] Zhao J., Wang H., Zhang Z., Zhou X., Yao J., Zhang R., Liao L., Dong J. (2019). Vitamin D deficiency as a risk factor for thyroid cancer: A meta-analysis of case-control studies. Nutrition.

[4] Kim D. (2016). Low vitamin D status is not associated with thyroid cancer risk. J. Endocrinol. Metab..

[5] Muscogiuri G., Tirabassi G., Bizzaro G., Orio F., Paschou S., Vryonidou A., Balercia G., Shoenfeld Y., Colao A. (2015). Vitamin D and thyroid disease: To D or not to D?. Eur. J. Clin. Nutr..

[6] Carlberg C., Muñoz A. (2022). An update on vitamin D signaling and cancer. Semin. Cancer Biol..

[7] National Cancer Institute (2013). Vitamin D and Cancer Prevention. https://www.cancer.gov/about-cancer/causes-prevention/risk/diet/vitamin-d-fact-sheet.

[8] Parva N.R., Tadepalli S., Singh P., Qian A., Joshi R., Kandala H., Nookala V.K., Cheriyath P. (2018). Prevalence of Vitamin D Deficiency and Associated Risk Factors in the US Population (2011–2012). Cureus.

[9] Darling A.L., Blackbourn D.J., Ahmadi K.R., Lanham-New S.A. (2021). Very high prevalence of 25-hydroxyvitamin D deficiency in 6433 UK South Asian adults: Analysis of the UK Biobank Cohort. Br. J. Nutr..

[10] Younis E. (2017). Oncogenesis of Thyroid Cancer. Asian Pac. J. Cancer Prev..

[11] Kondo T., Ezzat S., Asa S. (2006). Pathogenetic mechanisms in thyroid follicular-cell neoplasia. Nat. Cancer.

[12] La Vecchia C., Malvezzi M., Bosetti C., Garavello W., Bertuccio P., Levi F., Negri E. (2015). Thyroid cancer mortality and incidence: A global overview. Int. J. Cancer.

[13] Rahib L., Smith B.D., Aizenberg R., Rosenzweig A.B., Fleshman J.M., Matrisian L.M. (2014). Projecting cancer incidence and deaths to 2030: The unexpected burden of thyroid, liver, and pancreas cancers in the United States. Cancer Res..

[14] Siegel R.L., Miller K.D., Jemal A. (2020). Cancer statistics, 2020. CA Cancer J. Clin..

[15] Palacios C., Gonzalez L. (2014). Is vitamin D deficiency a major global public health problem?. J. Steroid Biochem. Mol. Biol..

[16] Cashman K.D., Sheehy T., O’Neill C.M. (2019). Is vitamin D deficiency a public health concern for low middle income countries? A systematic literature review. Eur. J. Nutr..

[17] Cashman K.D., Dowling K.G., Škrabáková Z., Gonzalez-Gross M., Valtuena J., De Henauw S., Moreno L., Damsgaard C.T., Michaelsen K.F. (2016). Vitamin D deficiency in Europe: Pandemic?. Am. J. Clin. Nutr..

[18] Nettore I.C., Colao A., Macchia P.E. (2018). Nutritional and Environmental Factors in Thyroid Carcinogenesis. Int. J. Environ. Res. Public Health.

[19] Morand G., Da Silva S.D., Hier M.P., Alaoui-Jamali M.A. (2014). Insights into genetic and epigenetic determinants with impact on vitamin D signaling and cancer association studies: The case of thyroid cancer. Front. Oncol..

[20] Jonklaas J., Danielsen M., Wang H. (2013). A pilot study of serum selenium, vitamin D, and thyrotropin concentrations in patients with thyroid cancer. Thyroid.

[21] Stepien T., Krupinski R., Sopiński J., Kuzdak K., Komorowski J., Lawnicka H., Stepien H. (2010). Decreased 1–25 Dihydroxyvitamin D3 concentration in peripheral blood serum of patients with thyroid cancer. Arch. Med. Res..

[22] Demircioglu Z.G., Aygun N., Demircioglu M.K., Yilmaz Ozguven B., Uludag M. (2022). Low vitamin D status is not associated with the aggressive pathological features of papillary thyroid cancer. Med. Bull. Sisli Etfal Hosp..

[23] Laney N., Meza J., Lyden E., Erickson J., Treude K., Goldner W. (2010). The prevalence of vitamin D deficiency is similar between thyroid nodule and thyroid cancer patients. Int. J. Endocrinol..

[24] Hu M.-J., Niu Q.-S., Wu H.-B., Lu X.-L., Wang L., Tong X.-R., Huang F. (2020). Association of thyroid cancer risk with plasma 25-hydroxyvitamin D and vitamin D binding protein: A case–control study in China. J. Endocrinol. Investig..

[25] Kuang J., Jin Z., Chen L., Zhao Q., Huang H., Liu Z., Yang W., Feng H., Yang Z., Díez J.J. (2022). Serum 25-hydroxyvitamin D level is unreliable as a risk factor and prognostic marker in papillary thyroid cancer. Ann. Transl. Med..

[26] Lopes N., Paredes J., Costa J.L., Ylstra B., Schmitt F. (2012). Vitamin D and the mammary gland: A review on its role in normal development and breast cancer. Breast Cancer Res..

[27] Colston K.W., Lowe L.C., Mansi J.L., Campbell M.J. (2006). Vitamin D status and breast cancer risk. Anticancer Res..

[28] Li Z., Wu L., Zhang J., Huang X., Thabane L., Li G. (2021). Effect of Vitamin D supplementation on risk of breast cancer: A systematic review and meta-analysis of randomized controlled trials. Front. Nutr..

[29] Balla B., Tobiás B., Kósa J.P., Podani J., Horváth P., Nagy Z., Horányi J., Járay B., Székely E., Krenács L. (2015). Vitamin D-neutralizing CYP24A1 expression, oncogenic mutation states and histological findings of human papillary thyroid cancer. J. Endocrinol. Investig..

[30] Kim M.J., Kim D., Koo J.S., Lee J.H., Nam K.H. (2022). Vitamin D Receptor Expression and Its Clinical Significance in Papillary Thyroid Cancer. Technol. Cancer Res. Treat..

[31] Guy M., Lowe L.C., Bretherton-Watt D., Mansi J.L., Peckitt C., Bliss J., Wilson R.G., Thomas V., Colston K.W. (2004). Vitamin D receptor gene polymorphisms and breast cancer risk. Clin. Cancer Res..

[32] McCullough M.L., Zoltick E.S., Weinstein S.J., Fedirko V., Wang M., Cook N.R., Eliassen A.H., Zeleniuch-Jacquotte A., Agnoli C., Albanes D. (2018). Circulating vitamin D and colorectal cancer risk: An international pooling project of 17 cohorts. J. Natl. Cancer Inst..

[33] Byers S.W., Rowlands T., Beildeck M., Bong Y.-S. (2012). Mechanism of action of vitamin D and the vitamin D receptor in colorectal cancer prevention and treatment. Rev. Endocr. Metab. Disord..

[34] Daremipouran M.R., Beyene D., Apprey V., Naab T.J., Kassim O.O., Copeland R.L., Kanaan Y.M. (2019). The association of a novel identified VDR SNP with prostate cancer in african american men. Cancer Genom. Proteom..

[35] Ntais C., Polycarpou A., Ioannidis J.P.A. (2003). Vitamin D receptor gene polymorphisms and risk of prostate cancer: A meta-analysis. Cancer Epidemiol. Biomarkers Prev..

[36] Beysel S., Eyerci N., Pinarli F.A., Apaydin M., Kizilgul M., Caliskan M., Ozcelik O., Kan S., Cakal E. (2018). VDR gene FokI polymorphism as a poor prognostic factor for papillary thyroid cancer. Tumor Biol..

[37] Nurminen V., Seuter S., Carlberg C. (2019). Vitamin D target genes of human monocytes. Front. Physiol..

[38] Hii C.S., Ferrante A. (2016). The Non-Genomic Actions of Vitamin, D. Nutrients.

[39] Feldman D., Krishnan A.V., Swami S., Giovannucci E., Feldman B.J. (2014). The role of vitamin D in reducing cancer risk and progression. Nat. Cancer.

[40] González-Duarte R.J., Cázares-Ordoñez V., Romero-Córdoba S., Díaz L., Ortíz V., Freyre-González J.A., Hidalgo-Miranda A., Larrea F., Avila E. (2015). Calcitriol increases Dicer expression and modifies the microRNAs signature in SiHa cervical cancer cells. Biochem. Cell Biol..

[41] Chang S.W., Lee H.C. (2019). Vitamin D and health—The missing vitamin in humans. Pediatr. Neonatol..

[42] Clinckspoor I., Verlinden L., Mathieu C., Bouillon R., Verstuyf A., Decallonne B. (2013). Vitamin D in thyroid tumorigenesis and development. Prog. Histochem. Cytochem..

[43] Davis C.D. (2008). Vitamin D and cancer: Current dilemmas and future research needs. Am. J. Clin. Nutr..

[44] Choi Y.M., Kim W.G., Kim T.Y., Bae S.J., Kim H.K., Jang E.K., Jeon M.J., Han J.M., Shong Y.K., Kim W.B. (2017). Serum Vitamin D3 Levels Are Not Associated with Thyroid Cancer Prevalence in Euthyroid Subjects without Autoimmune Thyroid Disease. Korean J. Intern. Med..

[45] Lee S., Morimoto S., Onishi T., Tsuji M., Okada Y., Seino Y., Ishida M., Yamaoka K., Takai S., Miyauchi A. (1982). Normal Serum 1,25-Dihydroxyvitamin D in Patients with Medullary Carcinoma of the Thyroid. J. Clin. Endocrinol. Metab..

[46] Ramezani M., Mazani M., Tabatabaei M., Rahimian A., Mosaferi E., Hedayati M. (2020). Medullary Thyroid Cancer Is Associated with High Serum Vitamin D Level and Polymorphism of Vitamin D Receptors. Physiol. Int..

[47] Deeb K.K., Trump D.L., Johnson C.S. (2007). Vitamin D signalling pathways in cancer: Potential for anticancer therapeutics. Nat. Rev. Cancer.

[48] Haussler M.R., Whitfield G.K., Kaneko I., Haussler C.A., Hsieh D., Hsieh J.C., Jurutka P.W. (2013). Molecular mechanisms of vitamin D action. Calcif. Tissue Int..

[49] Bikle D., Christakos S. (2020). New aspects of vitamin D metabolism and action—Addressing the skin as source and target. Nat. Rev. Endocrinol..

[50] Díaz G.D., Paraskeva C., Thomas M.G., Binderup L., Hague A. (2000). Apoptosis is induced by the active metabolite of vitamin D3 and its analogue EB1089 in colorectal adenoma and carcinoma cells: Possible implications for prevention and therapy. Cancer Res..

[51] McGuire T.F., Trump D.L., Johnson C.S. (2001). Vitamin D3-induced apoptosis of murine squamous cell carcinoma cells: Selective induction of caspase-dependent mek cleavage and up-regulation of Mekk-1. J. Biol. Chem..

[52] Abdel-Mohsen M.A., El-Braky A.A.-A., Ghazal A.A.E.-R., Shamseya M.M. (2018). Autophagy, apoptosis, vitamin D, and vitamin D receptor in hepatocellular carcinoma associated with hepatitis C virus. Medicine.

[53] Guarino V., Castellone M., Avilla E., Melillo R.M. (2010). Thyroid cancer and inflammation. Mol. Cell. Endocrinol..

[54] Liu W., Zhang L., Xu H.J., Li Y., Hu C.M., Yang J.Y., Sun M.Y. (2018). The Anti-Inflammatory Effects of Vitamin D in Tumorigenesis. Int. J. Mol. Sci..

[55] Perez-Fernandez R., Seoane S., Garcia-Caballero T., Segura C., Macia M. (2007). Vitamin D, Pit-1, GH, and PRL: Possible roles in breast cancer development. Curr. Med. Chem..

[56] Liu W., Asa S.L., Fantus I.G., Walfish P.G., Ezzat S. (2002). Vitamin D arrests thyroid carcinoma cell growth and induces p27 dephosphorylation and accumulation through PTEN/akt-dependent and -independent pathways. Am. J. Pathol..

[57] Dackiw A.P., Ezzat S., Huang P., Liu W., Asa S.L. (2004). Vitamin D3 administration induces nuclear p27 accumulation, restores differentiation, and reduces tumor burden in a mouse model of metastatic follicular thyroid cancer. Endocrinology.

[58] Carnero A., Blanco-Aparicio C., Renner O., Link W., Leal J.F. (2008). The PTEN/PI3K/AKT signalling pathway in cancer, therapeutic implications. Curr. Cancer Drug Targets.

[59] Dreijerink K.M., Varier R.A., van Nuland R., Broekhuizen R., Valk G.D., van der Wal J.E., Lips C.J., Kummer J.A., Timmers H.T. (2009). Regulation of vitamin D receptor function in MEN1-related parathyroid adenomas. Mol. Cell Endocrinol..

[60] An B.-S., Tavera-Mendoza L.E., Dimitrov V., Wang X., Calderon M.R., Wang H.-J., White J.H. (2010). Stimulation of Sirt1-regulated foxo protein function by the ligand-bound vitamin D receptor. Mol. Cell. Biol..

[61] Chiang K.C., Kuo S.F., Chen C.H., Ng S., Lin S.F., Yeh C.N., Chen L.W., Takano M., Chen T.C., Juang H.H. (2015). MART-10, the vitamin D analog, is a potent drug to inhibit anaplastic thyroid cancer cell metastatic potential. Cancer Lett..

[62] Fernández-Barral A., Bustamante-Madrid P., Ferrer-Mayorga G., Barbáchano A., Larriba M.J., Muñoz A. (2020). Vitamin D effects on cell differentiation and stemness in cancer. Cancers.

[63] Shah S., Hecht A., Pestell R., Byers S.W. (2003). Trans-repression of beta-catenin activity by nuclear receptors. J. Biol. Chem..

[64] Okda T.M., Abd-Εlghaffar S.K., Katary M.A., Abd-Alhaseeb M.M. (2021). Chemopreventive and anticancer activities of indomethacin and vitamin D combination on colorectal cancer induced by 1,2-dimethylhydrazine in rats. Biomed. Rep..

[65] Di Rosa M., Malaguarnera M., Nicoletti F., Malaguarnera L. (2011). Vitamin D3: A helpful immuno-modulator. Immunology.

[66] Karkeni E., Morin S.O., Tayeh B.B., Goubard A., Josselin E., Castellano R., Fauriat C., Guittard G., Olive D., Nunès J.A. (2019). Vitamin D controls tumor growth and CD8+ T cell infiltration in breast cancer. Front. Immunol..

[67] Bessler H., Djaldetti M. (2012). 1α,25-dihydroxyvitamin D3 modulates the interaction between immune and colon cancer cells. Biomed. Pharmacother..

[68] Hansen C.M., Binderup L., Hamberg K.J., Carlberg C. (2001). Vitamin D and cancer: Effects of 1,25(OH)2D3 and its analogs on growth control and tumorigenesis. Front. Biosci..

[69] Wacker M., Holick M.F. (2013). Sunlight and Vitamin D: A global perspective for health. Dermatoendocrinology.

[70] Grant W.B., Garland C.F. (2006). The association of solar ultraviolet B (UVB) with reducing risk of cancer: Multifactorial ecologic analysis of geographic variation in age-adjusted cancer mortality rates. Anticancer Res..

[71] Kricker A., Armstrong B. (2006). Does sunlight have a beneficial influence on certain cancers?. Prog. Biophys. Mol. Biol..

[72] Grant W.B., Juzeniene A., Moan J.E. (2011). Review Article: Health benefit of increased serum 25(OH)D levels from oral intake and ultraviolet-B irradiance in the Nordic countries. Scand. J. Public Health.

[73] Giovannucci E., Liu Y., Rimm E.B., Hollis B.W., Fuchs C.S., Stampfer M.J., Willett W.C. (2006). Prospective study of predictors of vitamin D status and cancer incidence and mortality in men. J. Natl. Cancer Inst..

[74] Janowsky E.C., Lester G.E., Weinberg C.R., Millikan R.C., Schildkraut J.M., Garrett P.A., Hulka B.S. (1999). Association between low levels of 1,25-dihydroxyvitamin D and breast cancer risk. Public Health Nutr..

[75] Feskanich D., Ma J., Fuchs C.S., Kirkner G.J., Hankinson S.E., Hollis B.W., Giovannucci E.L. (2004). Plasma vitamin D metabolites and risk of colorectal cancer in women. Cancer Epidemiol. Biomark. Prev..

[76] Jacobs E.T., Giuliano A.R., Martínez M.E., Hollis B.W., Reid M.E., Marshall J.R. (2004). Plasma levels of 25-hydroxyvitamin D, 1,25-dihydroxyvitamin D and the risk of prostate cancer. J. Steroid Biochem. Mol. Biol..

[77] Hutchinson M.S., Grimnes G., Joakimsen R.M., Figenschau Y., Jorde R. (2010). Low serum 25-hydroxyvitamin D levels are associated with increased all-cause mortality risk in a general population: The Tromsø study. Eur. J. Endocrinol..

[78] Giovannucci E., Reichrath J. (2008). Vitamin D status and cancer incidence and mortality. Sunlight, Vitamin D and Skin Cancer.

[79] Bertino J.R. (2016). Landmark study: The relation of solar radiation to cancer mortality in North America. Cancer Res..

[80] Garland C., Barrett-Connor E., Rossof A., Shekelle R., Criqui M., Paul O. (1985). Dietary vitamin D and calcium and risk of colorectal cancer: A 19-year prospective study in men. Lancet.

[81] Garland C., Garland F.C., Shaw E., Comstock G.W., Helsing K.J., Gorham E.D. (1989). Serum 25-hydroxyvitamin D and colon cancer: Eight-year prospective study. Lancet.

[82] Bertone-Johnson E.R., Chen W.Y., Holick M.F., Hollis B.W., Colditz G.A., Willett W.C., Hankinson S.E. (2005). Plasma 25-Hydroxyvitamin D and 1,25-Dihydroxyvitamin D and Risk of Breast Cancer. Cancer Epidemiol. Biomark. Prev..

[83] Freedman D.M., Dosemeci M., McGlynn K. (2002). Sunlight and mortality from breast, ovarian, colon, prostate, and non-melanoma skin cancer: A composite death certificate based case-control study. Occup. Environ. Med..

[84] Keum N., Lee D.H., Greenwood D.C., Manson J.E., Giovannucci E. (2019). Vitamin D supplementation and total cancer incidence and mortality: A meta-analysis of randomized controlled trials. Ann. Oncol..

[85] Goulão B., Stewart F., Ford J., MacLennan G., Avenell A. (2018). Cancer and vitamin D supplementation: A systematic review and meta-analysis. Am. J. Clin. Nutr..

[86] Lappe J.M., Heaney R.P. (2012). Why randomized controlled trials of calcium and vitamin D sometimes fail. Dermatoendocrinology.

[87] Tan M.L., Abrams S.A., Osborn D.A. (2020). Vitamin D supplementation for term breastfed infants to prevent vitamin D deficiency and improve bone health. Cochrane Database Syst. Rev..

[88] Avenell A., Mak J.C., O’Connell D. (2014). Vitamin D and vitamin D analogues for preventing fractures in post-menopausal women and older men. Cochrane Database Syst. Rev..

[89] Khaw K.T., Stewart A.W., Waayer D., Lawes C.M., Toop L., Camargo C.A., Scragg R. (2017). Effect of monthly high-dose vitamin D supplementation on falls and non-vertebral fractures: Secondary and post-hoc outcomes from the randomised, double-blind, placebo-controlled ViDA trial. Lancet Diabetes Endocrinol..

[90] Bischoff-Ferrari H.A., Dawson-Hughes B., Staehelin H.B., Orav J.E., Stuck A.E., Theiler R., Wong J.B., Egli A., Kiel D.P., Henschkowski J. (2009). Fall prevention with supplemental and active forms of vitamin D: A meta-analysis of randomised controlled trials. BMJ.

[91] Bischoff-Ferrari H.A., Willett W.C., Orav E.J., Lips P., Meunier P.J., Lyons R.A., Flicker L., Wark J., Jackson R.D., Cauley J.A. (2012). A pooled analysis of vitamin D dose requirements for fracture prevention. N. Engl. J. Med..

[92] Jolliffe D.A., Camargo C.A., Sluyter J.D., Aglipay M., Aloia J.F., Ganmaa D., Bergman P., Bischoff-Ferrari H.A., Borzutzky A., Damsgaard C.T. (2021). Vitamin D supplementation to prevent acute respiratory infections: A systematic review and meta-analysis of aggregate data from randomised controlled trials. Lancet Diabetes Endocrinol..

[93] Barbarawi M., Kheiri B., Zayed Y., Barbarawi O., Dhillon H., Swaid B., Yelangi A., Sundus S., Bachuwa G., Alkotob M.L. (2019). Vitamin D Supplementation and Cardiovascular Disease Risks in More Than 83,000 Individuals in 21 Randomized Clinical Trials: A Meta-analysis. JAMA Cardiol..

[94] Zheng C., He L., Liu L., Zhu J., Jin T. (2018). The efficacy of vitamin D in multiple sclerosis: A meta-analysis. Mult. Scler. Relat. Disord..

[95] Zorzella-Pezavento S.F.G., Mimura L.A.N., Denadai M.B., de Souza W.D.F., Fraga-Silva T.F.C., Sartori A. (2022). Is there a window of opportunity for the therapeutic use of vitamin D in multiple sclerosis?. Neural. Regen. Res..

[96] Bouillon R., Manousaki D., Rosen C., Trajanoska K., Rivadeneira F., Richards J.B. (2022). The health effects of vitamin D supplementation: Evidence from human studies. Nat. Rev. Endocrinol..

[97] Kaur J., Ferguson S.L., Freitas E., Miller R., Bemben D., Knehans A., Bemben M. (2019). Association of Vitamin D Status with Chronic Disease Risk Factors and Cognitive Dysfunction in 50–70 Years Old Adults. Nutrients.

[98] Morishita M., Ohtsuru A., Kumagai A., Namba H., Sato N., Hayashi T., Yamashita S. (2005). Vitamin D3 treatment for locally advanced thyroid cancer: A case report. Endocr. J..

